# Simultaneous Laparoscopic and Thoracoscopic Biopsy via a Single Skin Incision Using a Port‐Sharing Procedure in Infantile Neuroblastoma: A Case Report

**DOI:** 10.1111/ases.70064

**Published:** 2025-04-20

**Authors:** Tokuro Baba, Satoru Hamada, Hideki Sakiyama, Shinobu Kiyuna, Tokiko Oshioro, Masaaki Kuda, Satoshi Ieiri, Mitsuhisa Takatsuki

**Affiliations:** ^1^ Department of Digestive and General Surgery Graduate School of Medicine, University of the Ryukyus Kiyuna, Ginowan Japan; ^2^ Department of Child Health and Welfare Graduate School of Medicine, University of the Ryukyus Kiyuna, Ginowan Japan; ^3^ Department of Pediatric Surgery, Research Field in Medicine and Health Sciences, Medical and Dental Sciences Area, Research and Education Assembly Kagoshima University Kagoshima Japan

**Keywords:** MYCN amplification, neuroblastoma, pediatric surgery, port‐sharing procedure, urachal tumor

## Abstract

Neuroblastoma, typically presenting with atypical symptoms, rarely manifests as a urachal tumor. We report a case of urachal neuroblastoma wherein a single port site was used for both laparotomy and thoracoscopy. A 1‐month‐old female presented with umbilical discharge. Enhanced computed tomography revealed a urachal tumor, later confirmed pathologically as neuroblastoma without MYCN amplification. During chemotherapy for low‐risk classification, a right adrenal mass and mediastinal lymphadenopathy emerged, prompting further biopsy. We employed a port‐sharing procedure at the right hypochondrium to perform simultaneous laparoscopic and thoracoscopic biopsies, reducing the number of port‐side wounds. This approach could be applicable in other cases requiring combined thoracic and abdominal surgical access.

## 1 Introduction

Reduced port surgery has been increasingly used in pediatric surgical procedures due to its cosmetic benefits [1]. Although single‐port surgery is an ideal approach, it poses technical challenges, particularly in pediatric patients, due to limited working space. Multiple port incisions can be a relatively large for children although each wound is small. Reducing the number of ports in conventional port surgeries can also enhance cosmetic outcomes. We present a case of infantile neuroblastoma, presenting as a urachal tumor, where a laparoscopic and thoracoscopic biopsy was performed through a single port‐site incision.

## 2 Case Presentation

**FIGURE 1:**
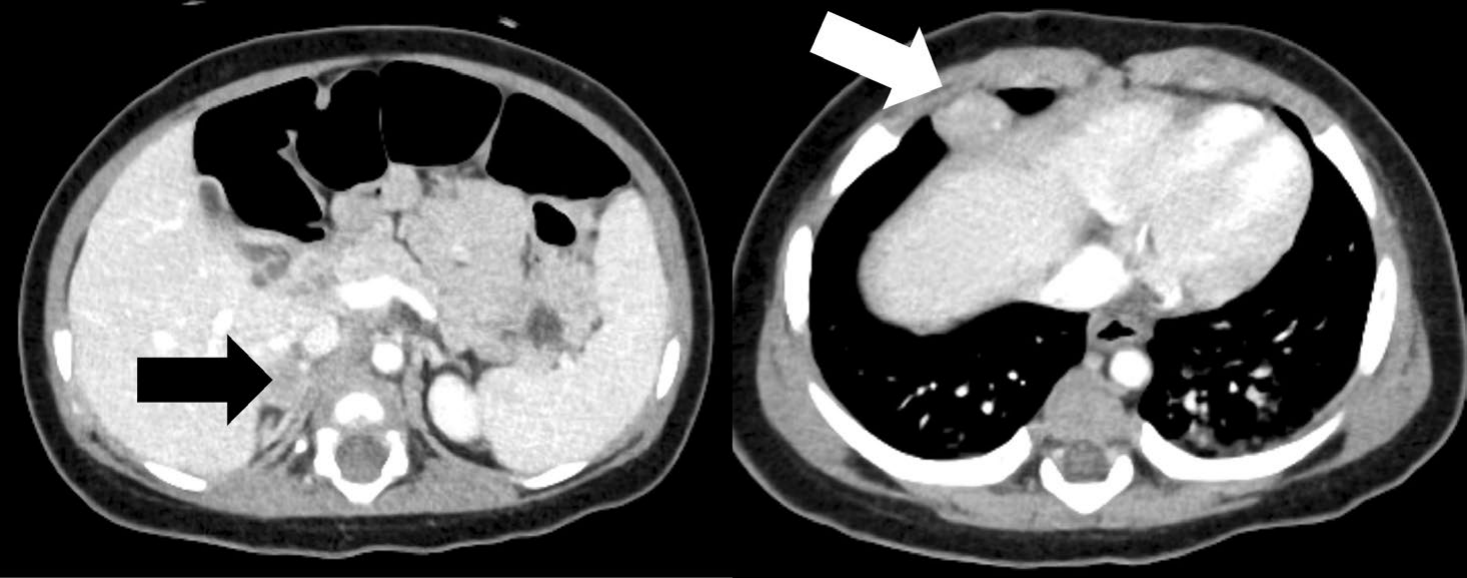
Computed tomography images at tumor progression. Left panel: The black arrow indicates the right adrenal mass. Right panel: The white arrow indicates the mediastinal lymph node.

**FIGURE 2:**
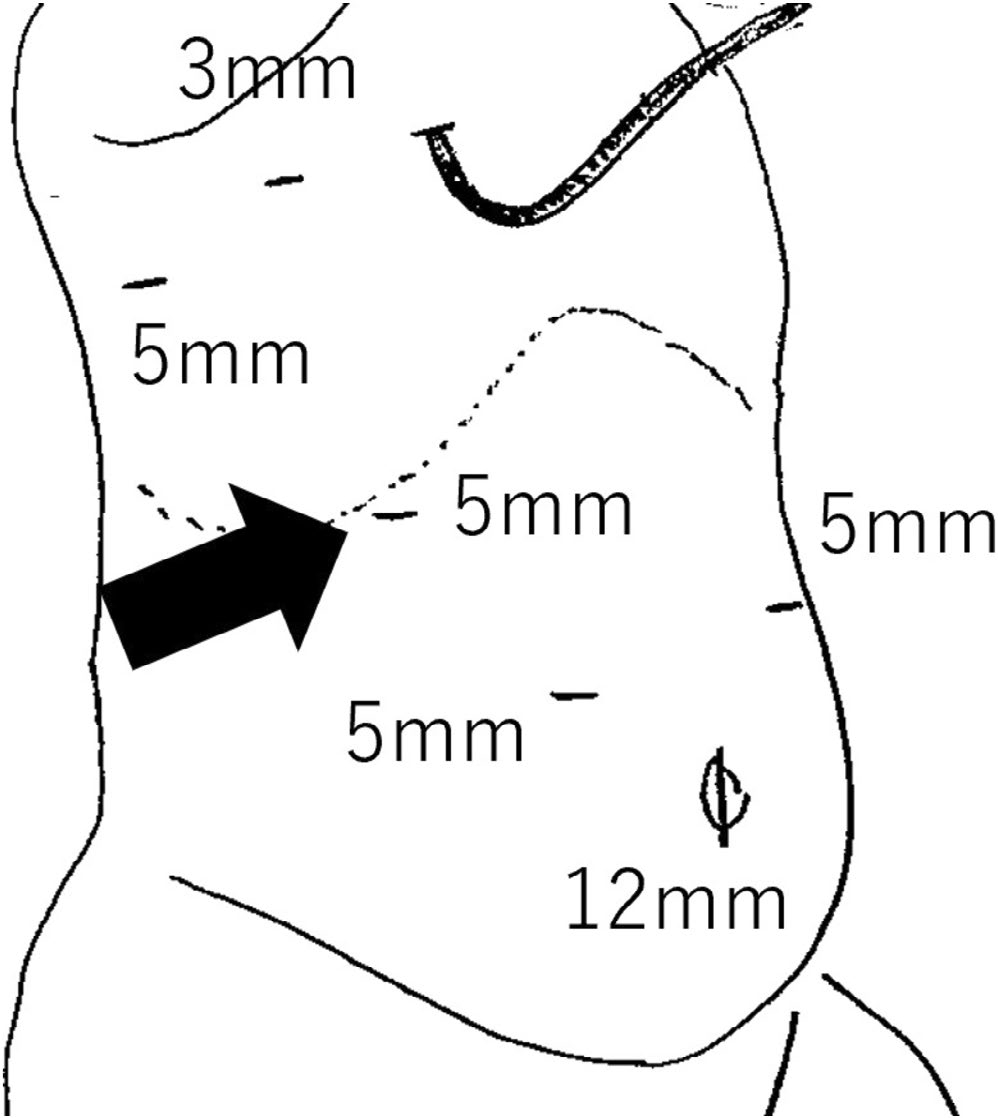
Port configuration for laparoscopic and thoracoscopic surgery. Black arrow indicates the port shared for both laparoscopic and thoracoscopic procedures.

A 1‐month‐old female presented with umbilical discharge and was referred to our hospital with a suspected urachal remnant. Enhanced computed tomography (CT) showed a tumor located at the bladder dome, connected to the umbilicus, with enlargement of bilateral iliac lymph nodes (Figures S1 and S2). No tumor was detected in the adrenal glands. Neuron‐specific enolase levels were slightly elevated at 40.4 ng/mL, with homovanillic acid (HVA) and vanillylmandelic acid (VMA) urine levels at 35.7 and 33.0 μg/mgCr, respectively. Resection of the urachal tumor was performed via open surgery. Partial cystectomy was required due to tumor invasion into the bladder muscle wall. Iliac lymph nodes were left in place without resection. Histopathological examination revealed a small round cells with abundant neurite formation and multiple Homer Wright‐type rosettes (Figure S3). The final diagnosis was poorly differentiated neuroblastoma with an intermediate mitotic kinetic index consistent with favorable histology according to the International Neuroblastoma Pathology Classification. MYCN amplification was absent. Bone marrow aspiration showed no evidence of tumor involvement, and the Iodine^123^‐labeled metaiodobenzylguanidine (I^123^ MIBG) scan was negative for lymphatic metastasis. Considering the primary urachal tumor and the image‐defined risk factor (IDRF)‐positive metastasis to the iliac lymph nodes, the International Neuroblastoma Risk Group (INRG) stage was determined to be L2. The patient was provisionally classified in the low‐risk category and was treated with vincristine and cyclophosphamide chemotherapy. After three cycles, a CT scan indicated worsened bilateral iliac lymph node metastasis, accompanied by a newly emerged right adrenal mass and additional mediastinal lymphatic metastasis (Figure 1). I^123^ MIBG showed mild uptake of the tracer activity in the paraaortic and iliac lymph nodes, but not in the right adrenal mass. To confirm the pathology of these new lesions, we scheduled biopsies using combined laparoscopic and thoracoscopic approaches. Under general anesthesia, the patient was positioned in the left semi‐lateral orientation. The port layout and intraoperative findings are shown in Figure 2 and Video S1, respectively. A 12‐mm trocar was inserted at the umbilicus using the open Hasson technique, and an 8 mmHg pneumoperitoneum was established with CO_2_ insufflation at 4 L/min. The operator's working ports were 5‐mm trocars placed bilaterally in the upper abdomen, and a 3‐mm trocar was positioned in the right hypochondrium for the assistant. After the assistant elevated the liver, the surgeon used a vessel‐sealing system (CoolSeal, BolderSurgical, Colorado, USA) to dissect and remove the right adrenal mass en bloc.

Thoracoscopic surgery followed. A 5‐mm trocar for the camera port was inserted via the postaxillary seventh intercostal space (ICS) using the optical technique, and artificial pneumothorax was induced with CO_2_ at 2 L/min. A 3‐mm trocar was placed in the midaxillary fifth ICS for the operator's left hand, and another 5‐mm trocar was inserted through the extended right hypochondrium incision for the operator's right hand. To minimize the risk of diaphragm injury, subcutaneous tissue was bluntly dissected using Kelly forceps, directing the wound cranially before port insertion. A mediastinal lymph node was easily identified and resected with the vessel‐sealing system (CoolSeal, BolderSurgical, Colorado, USA). Pathology revealed that the right adrenal mass and thoracic lymph node had identical features without MYCN amplification, matching the primary tumor's profile (Figure S4).

**FIGURE 3:**
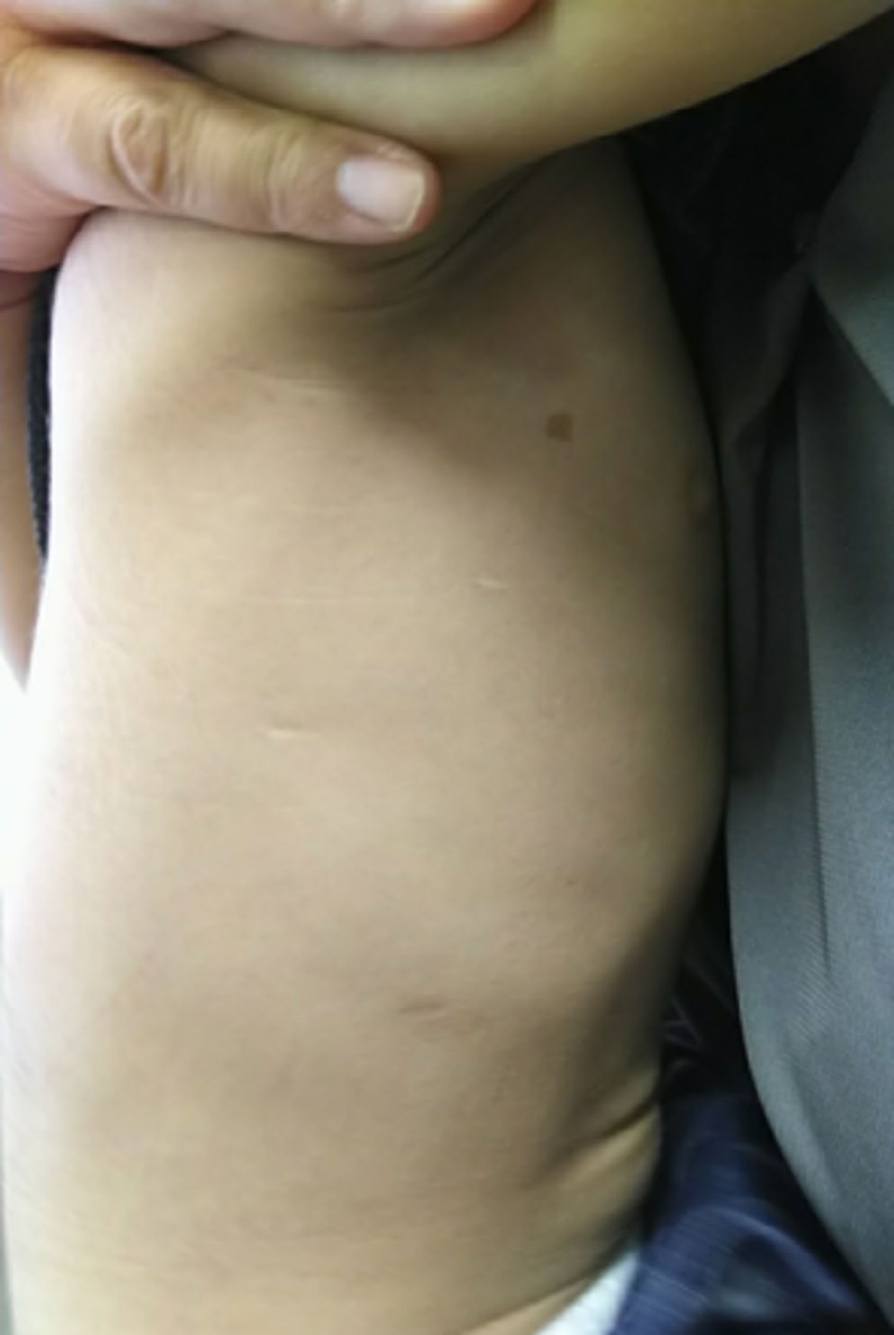
Appearance of the surgical wound 18 months after the second surgery.

Because the urachal tumor was identified as a metastatic lesion from the right adrenal neuroblastoma rather than a primary tumor, the patient was reclassified to the intermediate‐risk category. Her treatment was adjusted to an intermediate‐risk chemotherapy regimen comprising vincristine, cyclophosphamide, pirarubicin, and cisplatin. Tumor markers decreased to normal levels, and I^123^ MIBG uptake in the lymphatic lesions resolved following three cycles of dose‐intensive chemotherapy. An additional three cycles were administered, after which the final CT scan indicated shrinkage and calcification in the lymphatic lesions within the iliac and paraaortic regions. The patient has remained exacerbation‐free, with cosmetically satisfactory wounds 18 months post‐biopsy (Figure 3).

## 3 Discussion

For pediatric patients and their caregivers, cosmetic outcomes from surgical procedures are a significant concern, and surgeons should aim to minimize the number and size of surgical incisions. Several innovations have been documented in the literature of pediatric patients, including single‐port appendectomy through an umbilical incision [2] and single‐incision cholecystectomy [3], which consolidate multiple incisions into a single, cosmetically favorable site. However, these approaches are technically challenging, particularly in pediatric patients.

In our case, conventional port surgery would have required four abdominal and three thoracic port incisions, potentially compromising cosmetic outcomes even if each incision was small. Therefore, we opted to use a single port located in the right hypochondrium for both laparoscopic and thoracoscopic procedures, effectively reducing the number of incisions. This approach offers specific advantages for younger patients. First, in infants, the proximity of the thorax and abdomen allows for efficient access to both cavities from a single midpoint. Second, the elasticity and reduced thickness of an infant's skin make it more adaptable to such dual procedures [4]. We believe that this method can significantly improve cosmetic outcomes in pediatric surgeries and may be applicable in other cases requiring simultaneous thoracic and abdominal access.

Cases of neuroblastoma presenting as urachal tumors are extremely rare, with only one previously reported case [5, 6]. Generally, urachal tumors are asymptomatic until they reach a substantial size [7]. In this case, umbilical discharge provided an early diagnostic clue. In our case, the development of a right adrenal mass following initial low‐risk treatment suggests that heterochronous adrenal neuroblastoma may have been the primary tumor site. While conclusive data are limited, we hypothesize that the urachus is unlikely to serve as a primary neuroblastoma site.

## Author Contributions

T.B. wrote the manuscript. T.B., M.K., and S.I. performed the surgical procedures. S.H., H.S., S.K., and T.O. managed chemotherapy. S.I. and M.T. supervised the manuscript preparation. All authors reviewed and approved the manuscript.

## Disclosure

The authors declare no economic interests that could be perceived as influencing the content of this paper. Written informed consent was obtained from the parent of the patient for the publication of this Case Report.

## Conflicts of Interest

Dr. Satoshi Ieiri is an Editorial Board member of ASES Journal and a co‐author of this article. To minimize bias, he was excluded from all editorial decision‐making related to the acceptance of this article for publication.

## Supporting information


**Figure S1.** Computed tomography image at initial presentation. The white arrow indicates the urachal tumor.

**Figure S2.** Computed tomography image at initial presentation showing enlarged bilateral iliac lymph nodes (black arrow).

**Figure S3.** Histopathological analysis of the urachal tumor.

**Figure S4.** Histopathological analysis of the right adrenal mass.

**Video S1.** Video demonstrating the laparoscopic and thoracoscopic procedures.

## Data Availability

The data that support the findings of this study are available on request from the corresponding author. The data are not publicly available due to privacy or ethical restrictions.
