# Supplementary figures and images for: Simultaneous Laparoscopic and Thoracoscopic Biopsy via a Single Skin Incision Using a Port‐Sharing Procedure in Infantile Neuroblastoma: A Case Report
Source: Asian J Endosc Surg. 2025 Apr 20;18(1):e70064. doi: 10.1111/ases.70064 (PMC12010031; doi:10.1111/ases.70064)

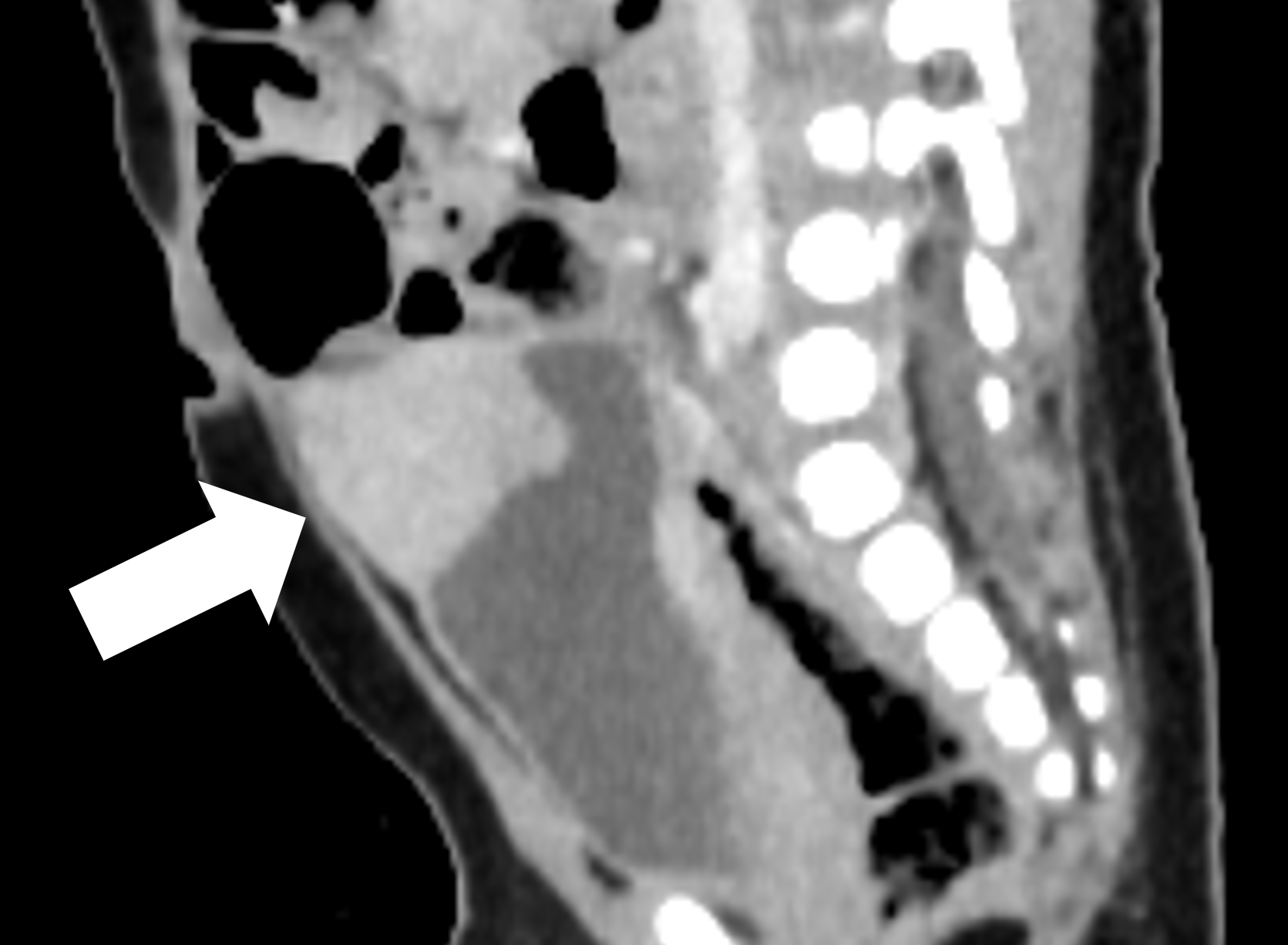

Supplement: Supplementary file 1 — Figure S1. Computed tomography image at initial presentation. The white arrow indicates the urachal tumor. [file ASES-18-e70064-s001.png]

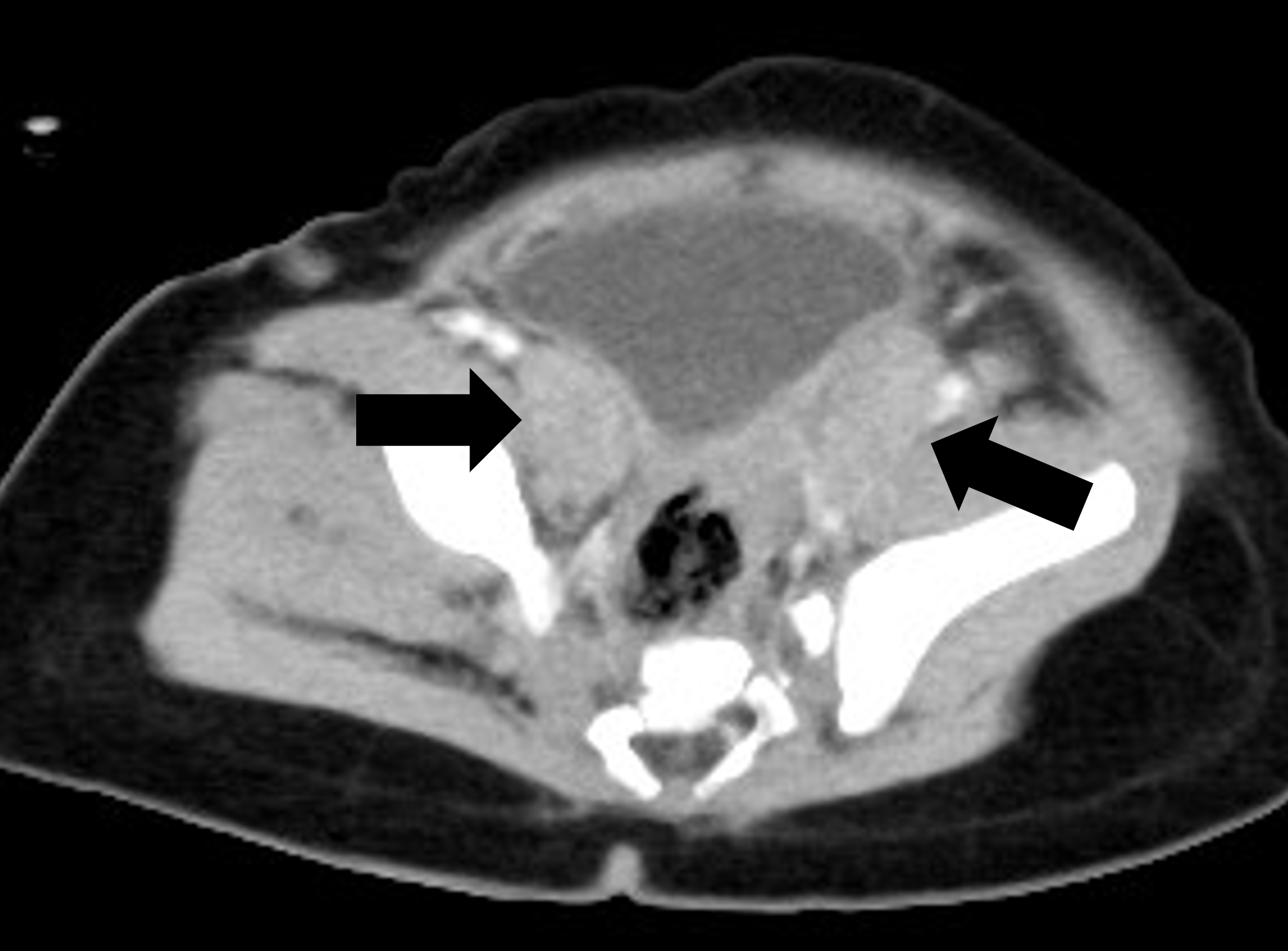

Supplement: Supplementary file 2 — Figure S2. Computed tomography image at initial presentation showing enlarged bilateral iliac lymph nodes (black arrow). [file ASES-18-e70064-s002.png]

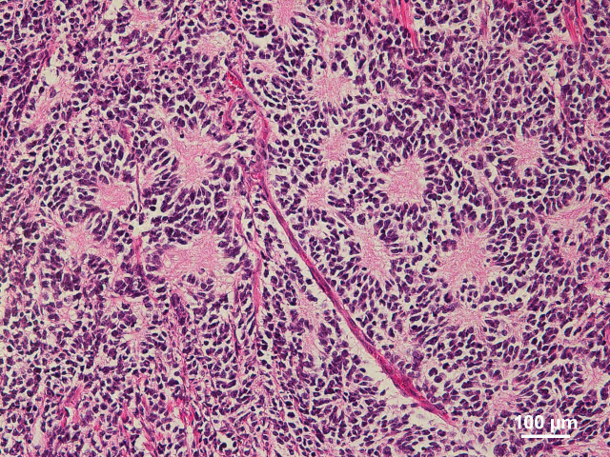

Supplement: Supplementary file 3 — Figure S3. Histopathological analysis of the urachal tumor. [file ASES-18-e70064-s003.png]

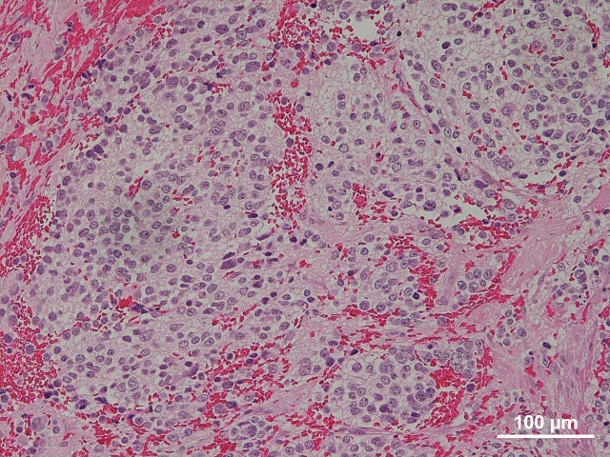

Supplement: Supplementary file 4 — Figure S4. Histopathological analysis of the right adrenal mass. [file ASES-18-e70064-s005.png]
